# Prevalence, characteristics and clinical impact of work-related musculoskeletal pain in echocardiography

**DOI:** 10.1186/s44156-024-00042-3

**Published:** 2024-03-06

**Authors:** Eunice Onwordi, Alistair Harris, Charlotte Atkinson, Cathy West, Keith Pearce, Jane Hancock, Camelia Demetrescu, Dhrubo Rakhit, Benoy N. Shah, Rajdeep Khattar, Jennifer Gorman, Delfin Encarnacion, Guy Lloyd, Sanjeev Bhattacharyya

**Affiliations:** 1 Bartholomew’s Hospital, West Smithfield, London, EC1A 7BE UK; 2 University Hospital Southampton Foundation NHS Trust, Southampton, UK; 3 Royal Brompton Hospital, London, UK; 4 Manchester University NHS Foundation Trust, Manchester, UK; 5 Thomas’ Hospital, London, UK; 6 University College London Hospitals, London, UK

**Keywords:** Echocardiography, Musculoskeletal injury, Work-related

## Abstract

**Background:**

Work-related musculoskeletal pain (WRMSP) is increasingly recognised in cardiac ultrasound practice. WRMSP can impact workforce health, productivity and sustainability. We sought to investigate the prevalence, characteristics and clinical impact of WRMSP.

**Methods:**

Prospective electronic survey of 157 echocardiographers in 10 institutions. Data acquired on demographics, experience, working environment/pattern, WRMSP location, severity and pattern, the impact on professional, personal life and career.

**Results:**

129/157 (82%) echocardiographers completed the survey, of whom 109 (85%) reported WRMSP and 55 (43%) reported work taking longer due to WRMSP. 40/129 (31%) required time off work. 78/109 (60%) reported sleep disturbance with 26/78 (33%) of moderate or severe severity. 56/129 (45%) required medical evaluation of their WRMSP and 25/129 (19%) received a formal diagnosis of musculoskeletal injury. Those with 11+ years of experience were significantly more likely to receive a formal diagnosis of WRMSP (p = 0.002) and require medication (p = 0.006) compared to those with 10 years or less experience.

**Conclusion:**

WRMSP is very common amongst echocardiographers, with a fifth having a related musculoskeletal injury. WRMSP has considerable on impact on personal, social and work-related activities. Strategies to reduce the burden of WRMSP are urgently required to ensure sustainability of the workforce and patient access to imaging.

**Supplementary Information:**

The online version contains supplementary material available at 10.1186/s44156-024-00042-3.

## What is already known on this topic?

There has been a sustained growth in the demand for echocardiography as the first line diagnostic test for nearly all suspected cardiovascular diseases. Work related musculoskeletal pain (WRMSP) has been reported in echocardiography. However, the prevalence, characteristics and clinical impact of WRMSP are poorly understood.

## What this study adds?

This study shows WRMSP is very common. A fifth of the respondents had a related musculoskeletal injury. WRMSP has a negative impact on work, productivity, sleep and well-being. WRMSP resulted in time off work and considering alternative employment due to pain. The number of years of scanning was related to increased rates of formal diagnosis of WRMSP and the need for therapy.

## How this study might affect research, practice or policy?

Urgent strategies to reduce WRMSP are required to ensure sustainability of the echocardiography workforce and to ensure that patients have access to diagnostic echocardiography.

## Background

There has been a worldwide increase in demand for cardiovascular imaging over the past couple of decades. Echocardiography is the most commonly performed investigation for assessment of cardiovascular structure and function. Echocardiography informs clinical decision making and is an essential diagnostic test in clinical practice.

Musculoskeletal injury or pain due to a work-related event is defined as a work-related musculoskeletal disorder (WRMD) [1, 2]. Work-related musculoskeletal pain (WRMSP) in cardiac sonographers has been described in the literature and is a common complaint [3]. Its consequences may include decreased workplace productivity, restriction in activities and a negative impact on wellbeing [3]. Furthermore, as demands for echocardiography grow, there may be a negative impact for patients due to a declining or debilitated workforce. It thus becomes increasingly important to recognise the impact of WRMSP on echocardiographers to drive solutions.

Studies in the United States (US) and Canada have brought attention to the problem of WRMSP in cardiac sonographers [3, 4]. There is wide variation in the echocardiography workforce, practice and protocols across the world. There are no data on the scale or impact from Europe. We sought to understand and characterise the frequency and impact of WRMSP in echocardiographers in 10 high volume centres in the United Kingdom.

## Methods

### Survey design and population

A survey was designed and administered through an electronic platform to cardiac sonographers/physiologists/cardiac scientists (echocardiographers) (n = 157) in 10 high volume centres in the United Kingdom. The participating centres included seven hospitals across London (St Bartholomew’s Hospital, Royal London hospital, Whipps Cross hospital, Newham University hospital, University College London Hospital, St Thomas’s Hospital & The Royal Brompton Hospital), University Hospital Southampton, Wythenshawe Hospital and Manchester Royal Infirmary. The survey was given institutional approval as a service evaluation. All respondents were anonymous.

Data were collected on echocardiographer demographics, experience, working environment and pattern. The survey also evaluated the proportion of time spent scanning, scanning style, physical activity, the presence and location of WRMSP, pain severity, the impact of pain on professional and personal life, and finally the influence of WRMSP on future career plans.

The survey was divided into the following sections:

1. Cardiac echocardiographer characteristics including age, gender, height, body weight, years of practice as a cardiac sonographer, scanning position, proportion of time spent scanning at work, preferred scanning hand and the presence of WRMSP.
2. The impact of pain at work and on activities of daily living including questions on pain severity, it’s effect on workplace productivity and recreational activities as well as sleep disturbance resulting from pain.
3. Interaction with medical services because of pain including the need for pharmacological therapy, physiotherapy or surgery as a result of WRMSP.
4. Formal medical diagnoses because of WRMSP.
5. The personal impact of WRMSP including financial difficulties because of time off work and/or strain on personal relationships. Participants were questioned as to whether they had considered finding alternative work because of the pain.

Pain was graded using the 11-point Numerical Rating Scale [5]. Echocardiographers were asked to score their pain on a scale of 0 (no pain at all), 1–3 (mild pain), 4–6 (moderate pain) and 7–10 (severe pain).

An 11-item questionnaire (The Disabilities of the Arm, Shoulder and Hand Outcome Measure (QuickDASH) was incorporated into the survey to assess both symptoms and function in cardiac sonographers with WRMSP in the upper limbs (Additional file 1). Each item within the QuickDASH questionnaire has five possible responses with a higher score reflecting a greater level of disability.

The survey was sent to participants in December 2022 and closed 5 weeks later in January 2023. All participants received weekly e-mail reminders to complete the survey. WRMSP was defined as pain that the echocardiographer had experienced that was caused by and/or exacerbated by echocardiography. Data regarding physician evaluation, medical diagnoses and the need for therapy (pharmacological, surgical and physical) as a result of WRMSP were collected.

### Statistics

Descriptive data are reported as numbers and percentages. Categorical variables were analysed using Chi Squared analysis. Statistical analysis was performed using SPSS Version 24 (SPSS Inc, Chicago, IL). A value of P < 0.05 was considered significant.

## Results

One hundred and twenty-nine out of 157 (82%) echocardiographers completed the survey. Table 1 summarises the baseline characteristics of the cohort. The majority were female 94/129 (72.9%). This is comparable with data from similar studies [3]. Nearly half (60/129–46.5%) were aged between 25 and 34 which is an accurate reflection of staff in the workforce. The majority 105/129 (81.4%) used their right hand to scan, with the remaining cohort using their left hand (9/129 (7%)) or either hand (15/129 (11.6%)). Just over half (67/129 (51.9%)) of echocardiographers reported regularly relaxing their handgrip whilst scanning. 81/129 (63%) reported undertaking regular exercise. Most echocardiographers reported sitting whilst scanning either on an extender attached to the bed (49/129 (38%)), on a chair or stool next to the patients’ bed (32/129 (24.8%)) or sitting next to the patient on the bed (19/129 (14.7%)). Only 29/129 (22.5%) reported routinely standing up whilst scanning.

**Table 1:** Echocardiographer characteristics

Characteristics	n = (%)
**Gender**	
Male	35/129 (27.1)
Female	94/129 (72.9)
**Age (years)**	
< 18	0
18–24	3/129 (2.3)
25–34	60/129 (46.5)
35–44	44/129 (34.1)
45–54	10/129 (7.8)
55–64	11/129 (8.5)
65+	1/129 (0.8)
**Work setting**	
Inpatient	14/129 (1.6)
Outpatient	2/129 (10.8)
Both inpatient and outpatient	113/129 (87.6)
**Experience (years)**	
0–5	37/129 (28.7)
6–10	33/129 (25.6)
11–20	42/129 (32.5)
20+	17/129 (13.2)

### Musculoskeletal pain

**Fig. 1:**
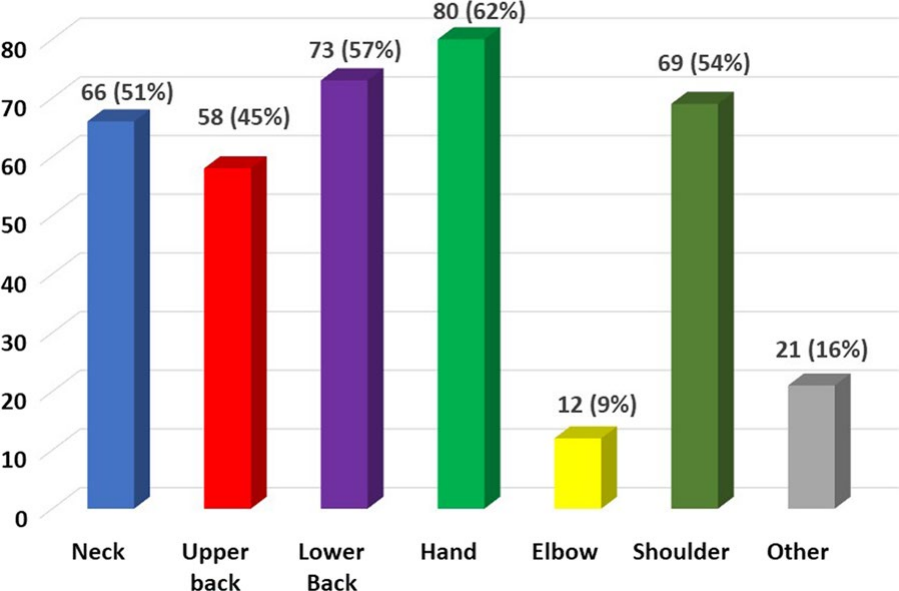
Location of work-related musculoskeletal disorders

**Fig. 2:**
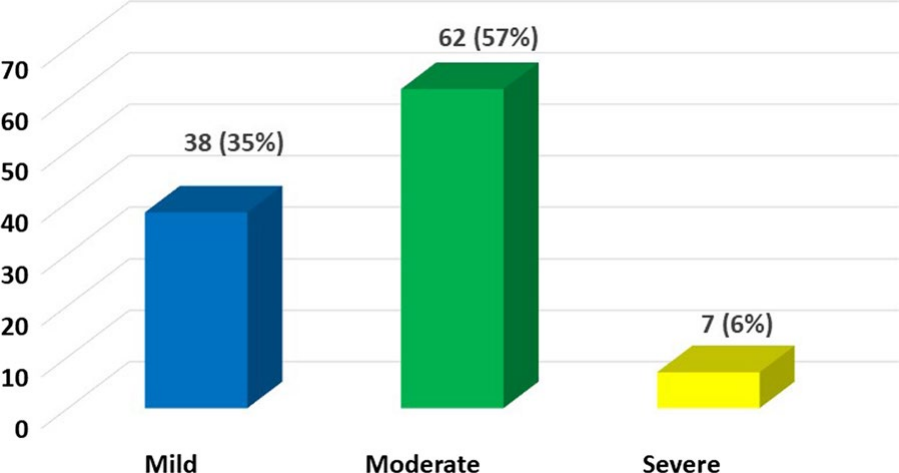
Numerical rating scale of severity of pain experienced on a weekly basis

WRMSP was common amongst respondents: 109/129 (85%). WRMSP was reported at multiple sites (Fig. 1). The most reported sites of pain were the hands (80/129 (62%)), the lower back (73/129 (56.6%)), shoulders (69/129 (53.5%)), neck (66/129 (51.2%)) and upper back (58/129, (45%)). Of those reporting hand pain 59/80 (74%) of this cohort reported pain in their right hand, 13/80 (16%) reported left hand pain and 8/80 (10%) reported experiencing pain in both hands. 107/109 completed the Numerical Rating Scale [5]. Pain severity was quantified as moderate or severe in 69/107 (64.5%) respondents (Fig. 2).

### Impact of WRMSP on clinical practice

WRMSP negatively impacted clinical practice. 96/129 (74%) reported experiencing a level of increased difficulty carrying out their work because of WRMSP. 98/129 (76%) reported that their WRMSP had a negative impact on their experience of work. 55/129 (43%) reported that their work took longer because of pain. Almost a third of the echocardiographers surveyed (40/129 (31%)) had required time off work due to WRMSP.

### Personal impact of WRMSP

Of the cohort, 56/129 (45%) required medical evaluation of their WRMSP by a physician. 25/129 (19%) received a formal medical diagnosis of musculoskeletal injury. Diagnoses included carpal tunnel syndrome, cervical spine, intervertebral disc or other spinal problems, rotator cuff injury and tennis elbow. 109/129 (85%) sought treatment for WRMSP. The most common forms of treatment included massage therapy (75/129, 58%) and pharmacological therapy with over-the-counter analgesia (73/129, 57%). Physiotherapy (60/129, 47%), topical analgesia (52/129, 40%) and prescription analgesia (20/129, 16%) were also used. 2 individuals required surgical procedures.

Sleep disturbance due to their WRMSP was reported in 78/129 (60%) with 26/78 (33%) of this cohort grading this sleep disturbance as moderate-severe. 86/129 (67%) reported experiencing WRMSP during recreational activities and 52/129 (40%) reported that their WRMSP had interfered with their normal social activities in the preceding week. 84/129 (65%) experienced pain whilst doing housework and 24/84 (29%) of this group graded this pain as moderate to severe.

A negative impact on personal relationships due to WRMSP was reported by 17/129 (13%) and 7/129 (5.4%) reported financial difficulties because of time off work due to WRMSP. 41/129 (32%) of the cohort had considered finding different employment due to WRMSP. 70/129 (54%) felt their employer did not have sufficient systems in place to prevent WRMSP.

### Age and pain

Overall, age was not significantly associated with reported WRMSP (p = 0.3) or severity of WRMSP (p = 0.53). Compared to younger peers (ages 18–24 and 25–34), sonographers aged between 35 and 44 were significantly more likely to have received a formal diagnosis of their WRMSP including neck, intervertebral disc or spinal problems, rotator cuff injuries, bursitis and carpal tunnel syndrome (p = 0.006). Use of over-the-counter analgesia, topical analgesia and prescription analgesia were significantly higher in those age 35–44 (p =  < 0.05) compared to those of younger age.

### Years of scanning and pain

As almost half of sonographers (45.7%) had 11+ years of experience we compared the incidence of WRMSP in those with 0–10 years of experience to those with 11+ years of experience. Those with 11+ years of experience were significantly more likely to have seen a physician due to WRMSP (p = 0.02) and received a formal diagnosis of their WRMSP (p = 0.002) including rotator cuff injury, bursitis or chronic headaches compared to those with 10 years or less experience. In addition, those with 11+ years of experience were also significantly more likely to have required analgesia both over the counter tablet medication (p = 0.006) and prescription analgesia (p = 0.004) to manage WRMSP.

## Discussion

To our knowledge, we carried out the first multi-institution survey of WRMSP and its impact on echocardiographers within Europe. Our findings potentially have major implications for both healthcare professionals and patients, since the prevalence of WRMSP amongst echocardiographers was extremely high (85%). WRMSP is associated with high morbidity including injuries, time off work & sleep disturbance. The greater number of years of scanning was related to increased rates of formal diagnosis of WRMSP and the need for therapy.

Barros-Gomes et al. [3] examined the impact of WRMSP at 10 Mayo Clinic facilities and identified WRMSP in 86% of sonographers, a prevalence consistent with our data. In our study, several different institutions with differing protocols were included. The number of echocardiograms performed per echocardiographer per day (7–10) are higher and duration of studies (45–60 min) are lower than the Mayo clinic (6 studies per day with a duration of 75 min). Therefore, although these factors may impact WRMSP, they are not the sole factors.

Although years of scanning (0–10 years vs 11+ years) was not significantly associated with reports of WRMSP or severity of WRMSP, there was a significant correlation between years of scanning and the need to seek physician evaluation for WRMSP, the need for analgesia to manage WRMSP and significantly higher rates of formal diagnosis of WRMSP including spinal problems and rotator cuff injury or bursitis and headaches. This suggests that WRMSP can develop early in a career, however the cumulative effects of WRMSP may build up over an echocardiographers career resulting in permanent injuries later in life. The impact of the total volume of scans performed in a lifetime may be a better metric to study in the future.

WRMSP had a negative impact on performance at work, ability to perform household tasks and social activities. Impact on work led to an increased time required to perform activities or time off work. Sleep disturbance, pain during housework or recreational activities were common. The result of this multi-faceted impact was associated with almost a third of respondents considering finding alternative employment due to WRMSP.

There is a paucity of evidence based research on methods to reduce or prevent WRMSP. Factors associated with WRMSP include ergonomics including machine, patient and sonographer factors. Collaboration with industry to address the weight and mechanics of the echo-console and ultrasound probe design should be encouraged. Expanding the capabilities of current point-of-care ultrasound systems (reducing the weight of echocardiographic equipment whilst maintaining standards) is a potential area of future development [6, 7]. Formal education in ergonomics including correct posture, hand grip and positioning of patients may help. Employers should pay attention to work schedules, facilitating and encouraging regular breaks to reduce the impact of repetitive strain [6]. Local exercise programmes including stretching prior to scanning may help to reduce the risk of WRMSP and injury [8]. Formal education at a local and national level regarding WRMSP could lead to earlier detection, earlier adjustment of work schedules and earlier interaction with physicians for appropriate treatment [9]. These measures could help to prevent progression and reduce the overall impact of WRMSP when it occurs.

## Survey limitations

The absence of a control group in this study is a limitation however such data is available in the literature. Barros-Gomes et al. demonstrated that echocardiographers are disproportionately impacted compared to peer employees [3].

The response rate of 82% was excellent. Echocardiographers with a history of WRMSP may have been more inclined to respond due to personal experience which may mean that those with pain are over-represented in this survey. The study relied on self-reported symptoms and impact and therefore may bias results. Prospective, multi-centre studies are required to evaluate mechanisms to reduce the occurrence of WRMSP.

## Conclusion

In this multi-centre study of WRMSP in echocardiographers, WRMSP was very common with a fifth having a related musculoskeletal injury. WRMSP had a negative impact on work, productivity, sleep and well-being. WRMSP resulted in time off work and considering alternative employment due to pain. The number of years of scanning was related to increased rates of formal diagnosis of WRMSP and the need for therapy.

### Supplementary Information

**Additional file 1.** Survey Questionnaire.

## Data Availability

The datasets used and/or analysed during the current study are available from the corresponding author on reasonable request.
