# Supplementary material for: Prevalence, characteristics and clinical impact of work-related musculoskeletal pain in echocardiography
Source: Echo Res Pract. 2024 Mar 6;11:6. doi: 10.1186/s44156-024-00042-3 (PMC10916016; doi:10.1186/s44156-024-00042-3)
Supplement: Supplementary file 1 — Additional file 1. Survey Questionnaire. [file 44156_2024_42_MOESM1_ESM.doc]

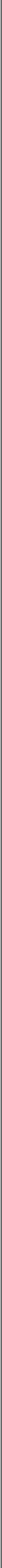

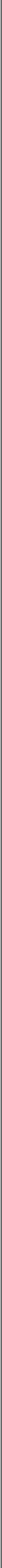
Characterisation


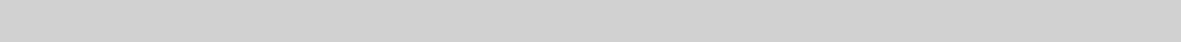


**Employee characteristics**

- - 1. How old are you


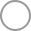
 Under 18


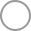
 18-24


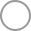
 25-34


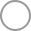
 35-44


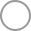
 45-54


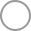
 55-64


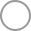
 65+

- - 2. What is your gender?


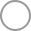
 Female


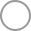
 Male


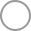
 Other (specify)

* 3. What is your height in centimetres?


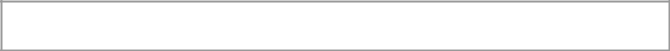

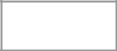


* 4. What is your current weight in kilograms?


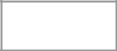


- 5. What is your usual work setting


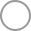
 Outpatient


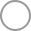
 Inpatient


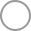
 A combination of outpatient and inpatient work

- 6. In what position do you normally scan?


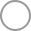
 Standing


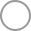
 Sitting on a chair/stool


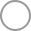
 Sitting on the bed/couch


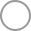
 Sitting on the bed/couch extender

-
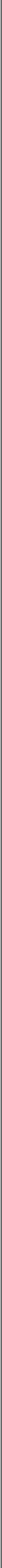

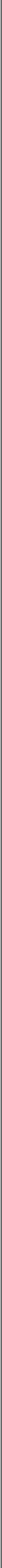
7. For how many years have you been practicing as an echocardiographer?


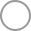
 0-5 years


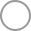
 6-10 years


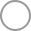
 11-20 years


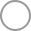
 20+ years

- 8. What proportion of your time at work is spent scanning?


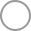
 < 25%


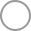
 25-50%


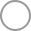
 51-75%


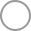
 >75%

- 9. In addition to your standard working hours do you provide an overnight or on-call echocardiography service as part of an on call rota?


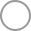
 Yes


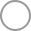
 No

- 10. Which hand do you use to scan?


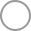
 Right


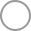
 Left


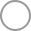
 Either - I am ambidextrous

- 11. Do you exercise regularly?


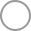
 Yes


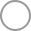
 No

- 12. Do you regularly relax your handgrip whilst scanning?


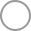
 Yes


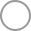
 No

- 13. Do you have physical pain that you attribute to echocardiography?


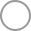
 Yes


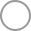
 No

- 14. Did your current pain issues exist prior to employment in echocardiography?


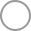
 Yes


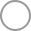
 No


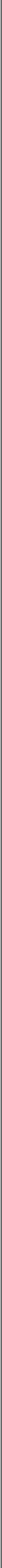

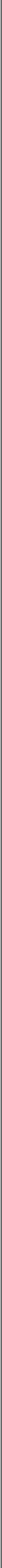
* 15. What was the location of your work-related pain? Please select all that apply?

Neck


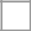


Upper back


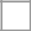


Lower back


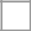


Hand


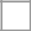


Elbow


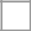


Shoulder


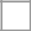


Other. Please specify


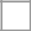


16. If you have hand pain in which hand was your pain located?


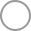
 Right


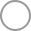
 Left


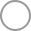
 Both

- 17. Which hospital do you work at?


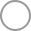
 Bart's Health


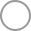
 St Thomas/Guys hospital


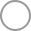
 Brompton


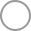
 Southampton


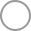
 Manchester University Foundation Trust


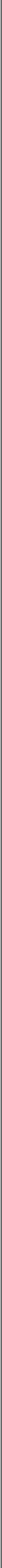

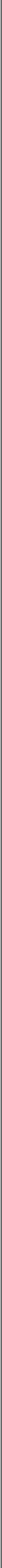
Impact


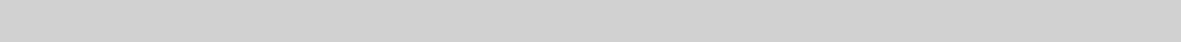


**Impact of the pain on daily living and work**

- 18. Please rate the intensity of pain you experience (on a scale of 0 to 10, 0 being no pain at all and 10 being extremely severe pain) on a weekly basis that is exacerbated by scanning.


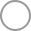
 0 - none


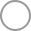
 1 - mild


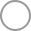
 2 - mild


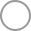
 3 - mild


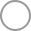
 4 - moderate


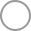
 5 - moderate


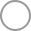
 6 - moderate


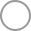
 7 - severe


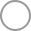
 8 - severe


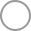
 9 - severe


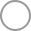
 10 - severe

- 19. How diﬀicult is it to work as a result of your pain?


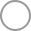
 No diﬀiculty


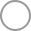
 Mild diﬀiculty


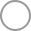
 Moderate diﬀiculty


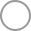
 Severe diﬀiculty


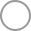
 Unable to work due to pain

- 20. How much of a negative impact does your pain have on the way you would like to work?


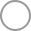
 No impact


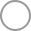
 Mild impact


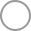
 Moderate impact


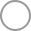
 Severe impact


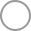
 I am now completely unable to work the way I want


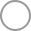
 I have no pain

-
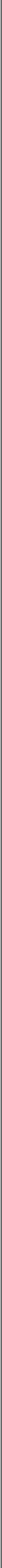

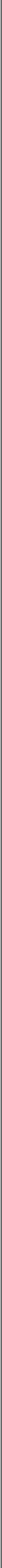
21. How much longer does it take you to do your work as a result of pain?


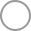
 No impact


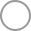
 It takes me slightly longer


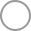
 It takes a lot longer


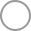
 I have no pain

- 22. Does your pain have any impact of pain on your sleep?


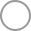
 No impact


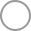
 Mild disturbance


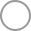
 Moderate disturbance


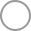
 Severe disturbance


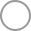
 I have no pain

- 23. How much pain do you experience during recreational activities


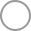
 None

a mild amount

a modest amount

severe pain

I can't do my normal activities because of the pain

- 24. How much pain do you experience during housework?

None

A mild amount

A modest amount

A severe amount

I can't do housework because of the pain

Interaction with medical services as a result of pain

- 25. Have you required medical evaluation by a physician as a result of the pain?

Yes

No

- 26. Have you needed surgical treatment to manage the pain

Yes

No

- 27. Have you needed over the counter pain tablet medication as a result of the pain?

Yes

No

- 28. Have you needed over the counter topical medication to manage the pain?

Yes

No

- 29. Have you needed prescription medication to manage the pain?

Yes

No

- 30. Have you needed physiotherapy to manage the pain?

Yes

No

- 31. Have you needed massage therapy to manage the pain?

Yes

No

- 32. Have you needed hot or cold therapy to manage the pain?

Yes

No

- 33. Have you been diagnosed with carpal tunnel syndrome since practicing echocardiography?

Yes

No

* 34. How have you funded payment treatment for the pain?

NHS treatment

Free prescriptions

Self-funded

Funded by employer

Not required - I have no pain

Diagnosis as a result of pain experienced

- 35. Has your pain resulted in a formal medical diagnosis?

Yes

No

N/A

- 36. Have you been diagnosed with a neck, intervertebral disc or spine problem since practicing echocardiography?

Yes

No

- 37. Have you been diagnosed with a cervical spine problem since working in echocardiography?

Yes

No

- 38. Have you been diagnosed with a thoracolumbar spinal problem since working in echocardiography?

Yes

No

- 39. Have you been diagnosed with a rotator cuﬀ injury or bursitis since working in echocardiography?

Yes

No

- 40. Have you been diagnosed with tennis elbow since working in echocardiography?

Yes

No

- 41. Have you been diagnosed with chronic headaches since working in echocardiography?

Yes

No

- 42. After how many years of working in echocardiography did you receive a formal medical diagnosis of your pain?

0-5 years

6-10 years

11-20 years

20+ years

- - 43. How much of your pain problems do you attribute to the practice of echocardiography?

My pain has nothing to do with the practice of echocardiography

A little

A lot

All of my pain has been caused by the practice echocardiography

I have no pain

* 44. Please rate your ability to do the following activities in the last week by selecting the appropriate response.

Moderate

No diﬀiculty Mild diﬀiculty diﬀiculty Severe diﬀiculty Unable

Open a tight or new

jar.

Do heavy household

chores (e.g., wash

walls, floors)

Carry a shopping

bag or briefcase.

Wash your back.

Use a knife to cut

food.

Recreational

activities in which

you take some force

or impact through

your arm, shoulder

or hand (e.g., golf,

hammering, tennis,

etc.).

Other (please specify)

- 45. During the past week, to what extent has your arm, shoulder or hand problem interfered with your normal social activities with family, friends, neighbours or groups?

Not at all

Slightly

Moderately

Quite a bit

Extremely

I have no pain

- 46. During the past week, were you limited in your work or other regular daily activities as a result of your arm, shoulder or hand problem?

Not at all

Slightly limited

Moderately limited

Very limited

Unable

- 47. Please rate the severity of the following symptoms in the last week.

None Mild Moderate Severe Extreme

Arm, shoulder or

hand pain

Tingling (pins and

needles) in your

arm, shoulder or

hand

- 48. During the past week, how much diﬀiculty have you had sleeping because of the pain in your arm, shoulder or hand?

No diﬀiculty

Mild diﬀiculty

Moderate diﬀiculty

Severe diﬀiculty

So much diﬀiculty that I can't sleep

Personal Impact

- 49. Have you had to take time oﬀ work as a result of pain?

Yes

No

- 50. Have you experienced financial diﬀiculty as a result of time oﬀ work due to pain?

Yes

No

- 51. Have your personal relationships suﬀered as a result of pain due to echocardiography?

Yes

No

- 52. Have you considered finding diﬀerent employment as a result of pain

Yes

No

Employer

- 53. Do you feel your employer has systems in place to prevent employee injury?

Yes

No
